# Leveraging sexually transmitted infection clinics for cervical cancer screening: evidence from India

**DOI:** 10.5588/pha.26.0036

**Published:** 2026-08-24

**Authors:** P. Yadav, A.A. Alwani, A.D. Harries, P.R. Deshmukh, J.P. Tripathy, N.B. Kuravatti, A. Dani, A. Pawar, M. Vijayageetha, K. Gavali, A. Seshachalam

**Affiliations:** 1 All India Institute of Medical Sciences (AIIMS), Nagpur, India;; 2 International Union against Tuberculosis and Lung Disease (The Union), Paris, France;; 3 The Union South-East Asia Office, New Delhi, India;; 4 London School of Hygiene and Tropical Medicine, London, UK;; 5 Tufts University School of Medicine, Boston, MA, USA;; 6 Maharashtra State AIDS Control Society (MSCACS), Mumbai, Maharashtra, India;; 7 Collaborative Medical Oncologist Group, India;; 8 Dr GVN Cancer Institute, Trichy, India.

**Keywords:** Pap smear, opportunistic screening, mixed-methods research, operational research, SORT-IT

## Abstract

**SETTING:**

Four government-run sexually transmitted infection/reproductive tract infection (STI/RTI) clinics at secondary and tertiary care hospitals in Nagpur district, Maharashtra, India.

**OBJECTIVE:**

To assess readiness and provider knowledge for Papanicolaou (Pap) smear–based cervical cancer screening (CCS), and to explore willingness, enablers, and barriers to CCS and integrating CCS into STI/RTI clinics.

**DESIGN:**

This was a convergent parallel mixed-methods study. Facility readiness and provider knowledge were assessed quantitatively. In-depth interviews explored implementation perspectives among purposively selected women clients, providers, and programme managers.

**RESULTS:**

Three out of four clinics met readiness standards, but only one clinic scored well in provider knowledge. Of 28 female clients, 27 (96%) were willing for CCS after counselling. Key enablers for clients to undergo CCS were respectful communication, and female-friendly and affordable services. Barriers included fear, embarrassment, low awareness, and poor family support. Providers (n = 9) highlighted well-equipped facilities, female staff availability, and permission from authorities to be key enablers for integration. They identified high workload and lack of training, staff, and programme-level support as constraints. The two programme managers perceived integration to be of less value.

**CONCLUSION:**

STI/RTI clinics could serve as a complementary platform for opportunistic CCS among high-risk women, provided human resources, training, awareness generation, and programme-level engagement are strengthened.

## Graphical abstract

**Figure:**
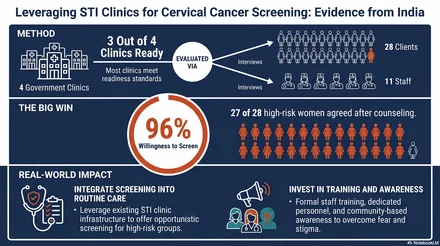

Cervical cancer remains one of the most preventable, deadly cancers affecting women, particularly in low- and middle-income countries (LMICs). In India, it is the second most common cancer among women,^1^ with an estimated 128,000 new cases and 80,000 deaths annually.^2^ Early detection through screening is crucial for preventing the progression of precancerous lesions to invasive cancer, and is known to reduce cervical cancer–related deaths by 80%.^3^ In 2016, India’s National Programme for Non-Communicable Diseases (NP-NCD) recommended universal cervical cancer screening (CCS) every 5 years for women aged 30 years or more at the primary care level.^4^ However, a decade after its implementation, CCS coverage remains suboptimal, similar to other LMICs with highest disease burden.^5–7^

Human papillomavirus is implicated in around 85% of cervical cancer cases,^8^ and is also the most common sexually transmitted infection (STI).^9^ This makes women attending designated STI/reproductive tract infection (RTI) clinics (DSRCs) a relevant high-risk group requiring screening. Integrating opportunistic CCS into existing services at DSRCs offers several potential advantages. First, it can increase access to screening^10^; second, DSRCs already engage women at high risk for cervical cancer; third, providers can offer a more comprehensive and streamlined approach to sexual and reproductive health by addressing multiple health concerns in a single visit; fourth, DSRCs may provide a less stigmatising environment for introducing CCS; and fifth, these facilities are equipped with necessary infrastructure and trained personnel. Leveraging this infrastructure could enhance CCS using Papanicolaou (Pap) smears without requiring the establishment of new stand-alone services and duplicate efforts.^11,12^ Pap smears have demonstrated higher specificity and accuracy for detecting precursor intraepithelial lesions and early signs of cervical cancer compared to visual inspection with acetic acid (VIA), although the latter has been adopted under the NP-NCD.^13^

Several studies have already demonstrated the feasibility and effectiveness of integrating CCS into reproductive health services. A scoping review revealed that CCS can be successfully integrated into primary care services, including STI clinics. Cost-effectiveness and strong leadership were reportedly important facilitators, while barriers included resource limitations and lack of national guidelines.^12^ Another study suggests that integrating CCS into family planning clinics or STI services can increase screening uptake and early cancer detection rates.^14^ This comes, however, with challenges including logistical difficulties in implementing new protocols in already crowded clinics, inadequate provider training, limited client awareness, and competing provider interests.^12^ Patient-related barriers like limited awareness, fear of diagnosis, and perceived complexity of multiple health issues may also hinder effective integration.^15^

A study analysing data from the National Family Health Survey-5 in India reported that the uptake of CCS was 1.9% among women aged 30–49 years, emphasising the need to improve screening coverage.^6^ Before attempting to integrate opportunistic CCS into DSRCs in India, it is important to assess facility readiness and understand the context-specific perspectives of key stakeholders. Thus, this study was undertaken with the following objectives in Nagpur district, India, in 2025, to: 1) assess the readiness of DSRCs for integrating CCS into routine care and provider knowledge for conducting Pap smears, 2) explore the willingness, enablers, and barriers for women clients attending DSRCs to undergo CCS at these clinics, and 3) assess the willingness, enablers, and barriers among medical officers (MOs), counsellors, laboratory technicians (LTs), and programme managers for DSRCs to integrate opportunistic CCS into routine care.

## METHODS

This study employed a convergent parallel mixed-methods design. We used a cross-sectional design to assess facility readiness and provider knowledge, and a descriptive qualitative approach to assess willingness, enablers, and barriers to CCS and its integration.

### Study setting

The study was conducted in four government-run DSRCs in Nagpur district, Maharashtra, Central India. The DSRCs were established by the National AIDS Control Organisation (NACO) and Maharashtra State AIDS Control Society (MSACS) to provide free, standardised STI/RTI services. These DSRCs were affiliated with secondary and tertiary care hospitals and had established pathways for testing and treating STIs and RTIs. Two facilities were affiliated with the same hospital and hence functioned independently as an STI clinic and an RTI clinic, while the other two provided comprehensive services. Staffing across DSRCs typically included an MO in charge and a counsellor; however, one facility was staffed solely by a male counsellor. An LT was available in only two facilities.

On average, 500–600 female clients were seen monthly at each DSRC except at the STI clinic where patients were predominately male. For patients presenting with symptoms of cervical cancer, one facility conducted CCS, while the other three did not. Instead, patients were referred to the Department of Gynaecology of the affiliated tertiary care hospital for testing.

### Study population

Objective 1: all four DSRCs, and the counsellors and LTs (n = 5) working there who could be designated to conduct Pap smears. Objective 2: all women aged 21 years or more who visited the DSRCs. Women with a history of cervical cancer were excluded. Consecutive sampling was used to include participants. When no new information was obtained from three consecutive interviews at one facility, data collection at the next facility was initiated. We interviewed 28 clients and achieved data saturation. Objective 3: 11 participants including all DSRC staff (three MOs, four counsellors, and two LTs) and one programme manager each from National AIDS/STI Control Programme (NACP) and NP-NCD.

### Data variables, data sources, and data collection

For Objective 1, we used the Improving Data for Decision Making in Global Cervical Cancer Programmes (IDCCP) Toolkit.^16^ This toolkit enables the calculation of an ‘Overall Facility Readiness Score’ using a framework of indicators. For this study, we adapted the toolkit to include seven relevant categories (with 37 items) to assess pre-implementation readiness. To score each facility, an investigator visited the facility, interviewed health care providers, observed workflow, and reviewed necessary documents. Scoring was performed as per the recommendations (0–2 for each item) and recorded on a paper-based form. Details of the scoring schedule and scoring guide are available here: https://figshare.com/s/cf04e9da6bdef48413c3?file=63336382.

To assess provider knowledge, an adapted version of the ‘Provider Skill’ component of the toolkit was used.^16^ The steps were reframed to assess knowledge regarding the steps of conducting a Pap smear. Each step was scored 0–2, using EpiCollect5.^17^ A score of 2 was given if all components of the step were enumerated; if some components were missed, a score of 1 was given. Details of the steps assessed and the scoring guide are here: https://figshare.com/s/43231af45dce05c0995b?file=63336379.

Each provider was also asked whether they had previously been trained to perform a Pap smear, had previously performed one, and how confident they were in conducting one.

For Objective 2, investigators conducted in-depth interviews with the clients from DSRCs, after obtaining informed consent, to explore willingness, enablers, and barriers to undergo CCS at the DSRC, using a semi-structured interview guide. As this was a sensitive topic, interviews were conducted by female doctors (PY, AD, AP). The interviews were conducted at a time convenient to participants at the clinic; privacy was ensured.

For Objective 3, doctors (PY, NK, AD, and AP) conducted and recorded key-informant interviews with programme managers telephonically after verbal consent, to understand their willingness, programmatic barriers, and enablers of integrating screening into DSRCs. Interviews were conducted with all the health care providers to understand their willingness and perspectives on enablers and barriers in implementing integrated opportunistic screening. The interviewers were fluent in local languages (Hindi and Marathi) and trained in qualitative research. Different semi-structured interview guides were used for each stakeholder group. The interviews were audio recorded and 15–45 min long. At the end of the interview, participants were debriefed and they verified a summary of their opinions.

### Analysis and statistics

For Objective 1: To calculate facility-wise Readiness Score, the average score for each category was manually calculated and then averaged to obtain an overall score. Thus, the total score ranged from 0 to 2. Average scores for each category and the overall readiness scores for all four DSRCs are reported. To calculate the provider knowledge score, data were extracted from EpiCollect5 onto Microsoft Excel and analysed. The average knowledge score for each provider and the average knowledge score for all facility providers were calculated and are reported. Interpretation of facility readiness score and provider knowledge score was as follows^16^: 1.8–2.0 = Meets the standard; 1.0–1.7 = Some improvement is needed to meet the standard; and 0.0–0.9 = Large improvement is needed to meet the standard. All quantitative findings are reported in line with Strengthening the Reporting of Observational Studies in Epidemiology (STROBE) guidelines.^18^

### Qualitative analysis for Objectives 2 and 3

Transcripts of the interviews were prepared in Marathi/Hindi using the audio recordings and translated to English on the same day of interview. Artificial intelligence–based tools (TurboScribe, Gemini) were used for transcription and translation. The outputs were reviewed and manually verified against the original audio recordings to ensure accuracy and completeness by PY, AD, NK, and AP. Two investigators (PY and AD) performed thematic analysis using ATLAS.ti 24 to identify codes using both deductive and inductive approaches on the willingness, enablers, and barriers to CCS and its integration in DSRCs. A third investigator (NK) reviewed the analysis, and the decision on coding and theme generation was made in consensus. The findings are reported in line with the ‘Consolidated Criteria for Reporting Qualitative Research’ (COREQ) guidelines.^19^

### Ethical statement

This study was conducted in accordance with the Declaration of Helsinki. Ethical approval was obtained from the Institutional Ethics Committee, All India Institute of Medical Sciences, Nagpur (IEC/Pharmac/2025/1552, dated 26 September 2025). Permission was obtained from MSACS before interviewing the participants (MSACS/Permission S.R.P/2025-26, dated 20 January 2026). Written or verbal informed consent was taken from all study participants.

## RESULTS

### Facility readiness and provider preparedness and knowledge

Assessment of the four DSRCs showed that three met the overall standards for facility readiness while one needed some improvement (Table 1). Across all four DSRCs, the domains infection prevention and referral mechanisms had top scores. In contrast, procurement and supply chain was the weaker domain in three facilities. Potential staffing was absent in one facility, causing that facility to not meet the readiness standards, as only one male counsellor was employed there and would not be designated to collect smears.

**TABLE 1.** Facility readiness scores for incorporating cervical cancer screening into routine care in designated sexually transmitted infection/reproductive tract infection clinics in Nagpur District, India, in 2025.

Facility readiness score categories	Number of items in each category	Average scores for each facility in relation to each category and overall			
		Facility 1	Facility 2	Facility 3	Facility 4
Potential staffing	1	2	2	0	2
Infrastructure	7	1.8	2	2	2
Procurement and supply chain	4	1.5	1.25	2	1.25
Equipment and supplies	7	1.6	2	2	2
Infection prevention	6	2	2	2	2
Data management	5	1.8	1.8	2	2
Referral mechanisms	7	2	2	2	2
Overall facility readiness average scores (0–2)		**1.8**	**1.9**	**1.7**	**1.9**

The ‘Improving Data for Decision Making in Global Cervical Cancer Programmes (IDCCP) Toolkit’ was used to assess the Facility Readiness Scores.^16^ The adapted version includes 37 items in 7 categories, each item scored as: ‘item is not present or is not functional’ – 0, ‘some interruptions in the presence and functioning of the item that affect quality of services’ – 1, and ‘item is present and functional on a regular and continuous basis or can be made so when required’ – 2. The average score for each category is calculated and presented. Interpretation of average scores: 1.8–2.0 = Meets the standard, 1.0–1.7 = Some improvement is needed to meet the standard, and 0.0–0.9 = Large improvement is needed to meet the standard. To determine the overall readiness score for a facility, all the category scores are summed and the average is calculated, so the total score for facility readiness ranges from 0 to 2. Detailed results are shown in https://figshare.com/s/cf04e9da6bdef48413c3?file=63336382.

Provider competence varied across the facilities (Table 2). Both providers in one facility, where Pap smears were conducted routinely, met the standards across training, experience, confidence, and knowledge. However, in another facility where Pap smears were not being conducted, despite both providers being trained and confident, they fared poorly when assessed for knowledge. One DSRC had one provider who lacked training, confidence, and knowledge, and another could not be assessed due to the absence of potential staff.

**TABLE 2.** Pap smear experience, confidence, and knowledge scores of care providers who may be designated to collect Pap smears in designated sexually transmitted infection/reproductive tract infection clinics in Nagpur District, India, in 2025.

	Total number of providers present who may have to collect a Pap smear **^A^**	Number of providers trained in collecting a Pap smear	Number of providers who ever collected a Pap smear	Number of providers who are somewhat or very confident in collecting a smear	Average knowledge scores of providers who may have to collect Pap smear (0–2)		
					Provider 1	Provider 2	Average score for the facility
Facility 1	2	2	2	2	1.4	0	**0.7**
Facility 2	2	2	2	2	2	2	**2**
Facility 4	1	0	0	0	0	NA **^B^**	**0**

Knowledge scoring of the care providers was adopted from the Provider Skill component of the ‘Improving Data for Decision Making in Global Cervical Cancer Programmes (IDCCP) Toolkit’.^16^ There are nine steps in performing the Pap smear, each of which was scored as: ‘Does not meet the knowledge criteria’ – 0, ‘Meets some knowledge criteria’ – 1 and ‘Meets the knowledge criteria’ – 2. At the end, for each provider, an average of these nine categories was taken to calculate the knowledge score.

Interpretation of the knowledge scores: 1.8–2.0 = Meets the standard, 1.0–1.7 = Some improvement is needed to meet the standard, and 0.0–0.9 = Large improvement is needed to meet the standard; since Medical Officers will not be designated to conduct Pap smears, their knowledge was not assessed.

Facility 3 had only one counsellor who was a male, so his experience, confidence, and knowledge were not assessed as he will not be expected to conduct Pap smears; additional details of each participant’s knowledge scores are shown in Annex 2 (https://figshare.com/s/43231af45dce05c0995b?file=63336379).

A Is the denominator for the following three columns.

B Facility 4 had only one staff, hence not applicable.

### Willingness, enablers, and barriers for cervical cancer screening amongst female clients

Twenty-eight female clients whose mean age (SD) was 33.3 (8) years were interviewed; 64% were homemakers and 86% had not previously undergone CCS (Table 3). Most were apprehensive about CCS due to poor awareness. However, after interviewers educated them about the benefits of screening, 27 (96%) expressed willingness to undergo CCS, while one of the clients refused. The prevalent belief was that early detection would lead to timely treatment and better outcomes. The one client who was unwilling to undergo screening believed that stigma was associated with the procedure and stated that, as she had no risk factors, she would not consider undergoing it.

**TABLE 3.** Socio-demographic and clinical characteristics of female clients visiting designated sexually transmitted infection/reproductive tract infection clinics (DSRCs) who were interviewed in Nagpur District, India (January 2026–February 2026).

Characteristics	Frequency (N), Percentage (%)
Total	28 (100)
Age (in years)	
21–30	10 (36)
31–40	12 (43)
41–50	4 (14)
51–60	2 (7)
Education	
No formal education	2 (7)
Primary	2 (7)
Secondary	15 (54)
Graduate	6 (21)
Post-graduate	3 (11)
Occupation	
Employed	9 (32)
Student	1 (4)
Home maker	18 (64)
Previous history of cervical cancer screening	
Yes	3 (11)
No	24 (86)
Cannot recall	1 (3)
Method of referral to DSRC	
Self-walk-in	22 (79)
Referred by a doctor	6 (21)

Women perceived CCS at DSRCs to be acceptable when offered in a trusted and respectful manner by doctors or counsellors. Enablers for the women included: good provider communication; easily accessible, female-friendly, and affordable services, including privacy and shorter wait times; and a clear understanding of the importance and process of CCS. Client-related factors, such as willingness, prior screening experience, symptoms, and knowledge of cervical cancer, were also described as enablers for CCS (Table 4). Perceived barriers to screening uptake included facility-level factors such as crowding at clinics and the need for repeat visits. Personal factors included lack of family support, embarrassment, low awareness, low perceived need, and competing responsibilities. Fear of pain and being diagnosed with a ‘dangerous disease’ were perceived to potentially hinder CCS uptake (Table 4).

**TABLE 4.** Enablers and barriers to cervical cancer screening with Pap smear in designated sexually transmitted infection/reproductive tract infection clinics (DSRCs) from female clients’ perspectives (January–February 2026).

Categories	Codes		Verbatims
Enablers			
Provider communication	• Doctors’ advice to undergo screening • Doctors personally explaining the need for the test and what will be done	• Trust in ASHAs **^A^** • ASHAs and Anganwadi workers’ advice • Good rapport with the counsellor	Yes, it should be done here itself. When we are already coming to the clinic, it becomes easier to do everything at the same place. – Client in HF3 I feel that the channels of the government, like there is an ad (advertisement) in the channels, if in that ad, if people get to know about cervical cancer, it will be very good. Like we see in movies, there is a lot of tobacco ads, detergent ads, there is one ad for cervical cancer, HIV, STI. – Client in HF4 It’s because it involves a private part of the body, so naturally there is some embarrassment. But I feel if we trust doctors during pregnancy and operations, then why should we feel ashamed for this test? – Client in HF1
Service accessibility and affordability	• All facilities available under one roof • Availability of a female doctor • Availability of privacy • Comfortable in seeking care at DSRCs • Nearby facility	• Less wait time compared to the gynaecology OPD • Free or low-cost screening/treatment • Suggestion: Dedicated OPD day for screening	
Good communication	• Including family in counselling • Awareness spread by cancer survivors • Explaining the importance and process of screening through personal interactions	• Spreading awareness through community-based programmes, social media, videos, posters, and advertisements • Prioritising patient’s comfort • Respectful behaviour of staff	
Client factors	• Client willing for CCS • Compliant patients • Presence of symptoms	• Underwent Pap smear previously • Knowledge about cervical cancer and its screening	
Barriers			
Facility-level factors	• Crowd at the clinic	• Requires multiple visits for reports	
Personal factors	• Role of family in health-related decisions • Fear (of pain, internal examinations, and diagnosis) • Lack of awareness	• Embarrassment • No perceived necessity • Too busy with other responsibilities • Stigma	

A ASHA = Accredited Social Health Activist, the term used for community health workers in India; OPD = outpatient department; CCS = cervical cancer screening; HIV = human immunodeficiency virus; STI = sexually transmitted infection; HF = health facility.

Family influence emerged as an important factor in decision making. Women reported that support from husbands or other relatives could facilitate screening, while its absence could prevent it.

### Willingness, enablers, and barriers to integrating cervical cancer screening into routine care in DSRCs

Three MOs, four counsellors, two LTs, and two programme managers were interviewed. Their median (interquartile range) duration of service was 16 years (11–19). Two of the three MOs were willing for integration of CCS into routine care, while the third believed it was better delegated to the department of gynaecology. The counsellors and LTs believed that although beneficial for clients, integrating CCS would add to their already high workload and would be better implemented if dedicated staff were recruited. Programme managers were not in favour of integrating CCS into DSRCs.

Key enablers for integrating CCS into DSRC services, as perceived by the providers, were availability of female staff, equipment, and administrative support (Table 5). Among counsellors and LTs, prior experience with cervical swab collection was an additional enabler. For MOs, orders from authorities to conduct CCS were considered important. However, several important operational barriers also emerged, including high workload, lack of dedicated private space, time constraints, staff shortages, and lack of formal training (Table 5).

**TABLE 5.** Enablers and barriers to integrating cervical cancer screening with Pap smear into routine care in designated sexually transmitted infection/reproductive tract infection clinics (DSRCs) from providers’ perspectives (January–February 2026).

Stakeholders	Codes	Verbatims
Enablers		
Medical officers Counsellors Lab technicians	• Permission/order from the authorities Provider-related: • Already taking cervical swabs • Availability of female staff to take smears • Experience of conducting Pap smears Facility-related: • Conducting screening as part of projects • Good supply chain • Well-equipped facility	If there were men in the OBGY OPD, patients wouldn't do it. So preferably, a female doctor or whatever technician or nurse is there should be female – Counsellor If the in-charge has said that it has to be done, then we will do it. – MO
Programme managers	NP-NCD: • Willingness of staff at DSRCs to conduct CCS	
Barriers		
Medical officers Counsellors Lab technicians	Provider-related: • Lack of time • No formal training • High workload • Shortage of staff Facility-related: • Lack of dedicated space	Besides that, if you talk about record-keeping and all those things – there is already so much work for a single person within our own project that we aren't even able to complete that; we are overstretched. – Counsellor
Programme managers of NP-NCD and NACP	• VIA already being done at PHCs • DSRCs are very few and far • Burden on the programme • Resource intensive	It is already being done at the PHCs…DSRCs are so few how will it help? – PM-NP-NCD

NP-NCD = National Programme for Non-Communicable Diseases; NACP = National AIDS Control Programme; VIA = visual inspection with acetic acid; PHC = primary health centre; PM = programme manager; OBGY = obstetrics and gynaecology; OPD = outpatient department; MO = medical officer.

From the perspective of the NP-NCD manager, VIA screening was already being done at primary health centres (PHCs), and DSRCs were considered too few to contribute substantially. While willingness of DSRC staff to conduct screening was considered as a potential enabler, CCS at DSRCs was not considered to offer programmatic value. The NACP manager considered CCS integration as potentially challenging and resource-intensive for the programme, requiring additional instruments, doctors, space, and staff. Overall, programme-level respondents questioned the added programmatic value of integrating CCS into DSRCs.

## DISCUSSION

This is the first study from India to provide evidence on the readiness of DSRCs for integrating opportunistic CCS, and the enablers and barriers to its implementation from providers’ perspectives. Additionally, the willingness of high-risk women to undergo CCS, along with associated enablers and barriers, was explored.

Our study found that DSRCs in Nagpur were ready to roll out opportunistic CCS, with the necessary infrastructure, supplies, and referral mechanisms in place. There was also consensus among providers at DSRCs that incorporating CCS would benefit patients. However, staff who would be directly involved in screening, that is, counsellors and LTs, expressed concerns about workload, time constraints, and lack of training. In the quantitative assessment too, shortcomings noted were trained human resources and inventory management, which are common challenges faced in LMICs.^12,20,21^ Integrating CCS into routine workflows at DSRCs would require structured training programmes, increased manpower, and streamlined processes. Programme managers were more sceptical about the benefits of integration given the existing programmatic burden and screening already being done by VIA at PHCs. However, almost 96% of high-risk women in our study were willing to undergo CCS in DSRCs, thereby indicating a high unmet need for it. Hence, efforts should be made to engage programme managers, as previous studies have identified high-level support to be an essential enabler of successful integration.^10,12,14^

Clients visiting DSRCs, despite being at high risk of cervical cancer, had poor awareness of CCS and their risk of cancer; only three had previously undergone screening. Similar findings have been consistently reported from other parts of India as well.^6,22^ After receiving counselling, one woman still refused to undergo screening, reflecting the stigma surrounding CCS. Such stigma may stem from beliefs that undergoing testing implies promiscuous behaviour and is therefore shameful.^23^ However, the 27 other women in our study, after being educated by the interviewers about the benefits of the procedure, were willing to undergo CCS at DSRCs. While this may have been influenced by social desirability bias, previous evidence from Maharashtra also demonstrated a favourable attitude and high uptake when CCS was offered to urban and tribal women, despite poor knowledge.^24^ Such findings emphasise that awareness generation is needed for increasing the willingness among women, and also underscore the importance of providing them with timely and accessible CCS.

The barriers to CCS perceived by our study clients, including poor awareness, embarrassment, fear, and low perceived need, are in line with previous literature from India and elsewhere.^5,12,25^ These may be addressed through peer and provider-led counselling, educational programmes, and the availability of female staff.^14,26^ The importance of family involvement in health-related decision making was also highlighted. This aligns with existing evidence that women’s health-seeking decisions in India are shaped by household dynamics,^5,27^ emphasising the need for family-sensitive counselling and community-based messaging for CCS uptake.

Given the unmet need for CCS among this population, incorporating screening into DSRCs is necessary from a patient-centred care perspective. This would serve as a targeted, complementary strategy to primary care–based screening for women already accessing STI/RTI services, provided gaps like staff shortages and low willingness of programme managers are addressed. Willingness of clients to undergo CCS will have to be addressed through awareness-generation activities and addressing stigma relating to CCS. Further implementation research could explore the feasibility of dedicating 1 day/ week to screening, as suggested by a client. A phased pilot approach in this district, jointly implemented by NACP and NP-NCD, starting with DSRCs that meet readiness standards and have high provider knowledge scores, could be considered going forward to assess the feasibility of integration.

The strengths of this study include the use of data triangulation^28^ by employing a mixed-methods design, and obtaining perspectives from all stakeholders, which provided actionable insights and comprehensive understanding of enablers and barriers. However, there were limitations. First, social desirability bias may have influenced responses and counselling increased client willingness. Additionally, the study was conducted in only one district, which may limit generalisability to other settings. Lastly, provider knowledge scores may have been deflated in the two facilities that did not meet the standards, as the tool administered included a score limiting step.

## CONCLUSION

DSRCs have the potential to serve as a complementary platform for opportunistic CCS among high-risk women. Engaging programme managers, strengthening human resources, and generating community awareness are essential for its effective implementation.
